# The Separation of Blood Components Using Standing Surface Acoustic Waves (SSAWs) Microfluidic Devices: Analysis and Simulation

**DOI:** 10.3390/bioengineering4020028

**Published:** 2017-03-29

**Authors:** Ahmed M. Soliman, Mohamed A. Eldosoky, Taha E. Taha

**Affiliations:** 1 Biomedical Engineering Department, Faculty of Engineering, Helwan University, Cairo11792, Egypt; mohamed_eldesouky@h-eng.helwan.edu.eg; 2 Communication Department, Faculty of Electronic Engineering, Menoufia University, Menouf 23952, Egypt; taha117@hotmail.com

**Keywords:** surface acoustic waves, microfluidics, separation of blood components, biomedical applications, analysis and simulations

## Abstract

The separation of blood components (WBCs, RBCs, and platelets) is important for medical applications. Recently, standing surface acoustic wave (SSAW) microfluidic devices are used for the separation of particles. In this paper, the design analysis of SSAW microfluidics is presented. Also, the analysis of SSAW force with Rayleigh angle effect and its attenuation in liquid-loaded substrate, viscous drag force, hydrodynamic force, and diffusion force are explained and analyzed. The analyses are provided for selecting the piezoelectric material, width of the main microchannel, working area of SAW, wavelength, minimum input power required for the separation process, and widths of outlet collecting microchannels. The design analysis of SSAW microfluidics is provided for determining the minimum input power required for the separation process with appropriated the displacement contrast of the particles.The analyses are applied for simulation the separation of blood components. The piezoelectric material, width of the main microchannel, working area of SAW, wavelength, and minimum input power required for the separation process are selected as LiNbO_3_, 120 μm, 1.08 mm^2^, 300 μm, 371 mW. The results are compared to other published results. The results of these simulations achieve minimum power consumption, less complicated setup, and high collecting efficiency. All simulation programs are built by MATLAB.

## 1. Introduction

White blood cells (WBCs), red blood cells (RBCs), and platelets are the components of blood. The separation of blood components is important for medical treatment and diagnosis. The standing surface acoustic wave (SSAW) microfluidic devices are positively significant in the separation of particles especially in biomedical applications. The separation of particles is depending on volume, density, and compressibility of the particles. The separation of particles using SSAW microfluidic devices has many advantages such as: miniaturization, high accuracy, high sensitivity, portable, low cost, low time consumption, and low samples consumption.

The surface acoustic wave (SAW) is generated by applying alternating electric (AC) signal to interdigital transducers (IDTs) based on the piezoelectricity phenomenon. IDTs are metal electrodes deposited on the piezoelectric substrate. The acoustic waves are launched and propagated away from and perpendicular to IDTs fingers. The wavelength is determined by the IDT geometry as following [1]:

*λ* = 4*d* (1)

where: *d* is the finger width of IDTs. The acoustic wave propagation velocity is determined by the acoustic mode, orientation and the type of substrate. The fundamental resonant frequency is related to the acoustic wave velocity as given as following:

(2) $$f_{0}=v_{0}/\lambda$$

where: *f*_0_, *v*_0_, and *λ* are the unperturbed frequency, the unperturbed velocity and wavelength of SAW, respectively. The interaction between SAWs and the medium can be used as sensors [2,3] or as biosensors in biological applications [4]. In our previous work [5,6], the principles of mathematical models of using SSAW microfluidic devices in the separation process were described and analyzed.

**Figure 1:**
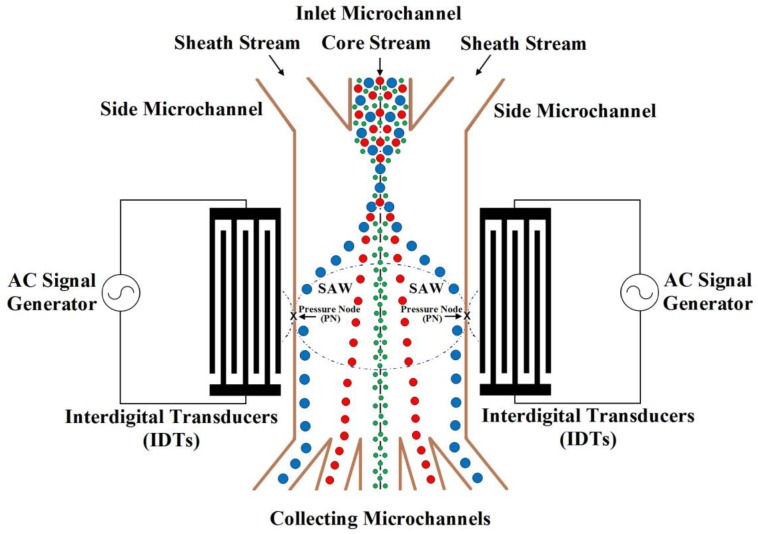
The schematic of SSAW microfluidic device for particles separation. (The figure is not to scale.)

The schematic of SSAW microfluidic device for particles separation is shown in Figure 1. The figure is not to scale. In this schematic, the particles mixture is flowed in an inlet microchannel among symmetric side microchannels. These flows produce hydrodynamic force. Also, the SSAW force can migrate the particles to the pressure node or antinode depending on its acoustic contrast factor sign as will be explained later. The particles are collected by outlet collecting microchannels according to its physical properties.

In Reference [7], the separation of three different sizes (1, 5, and 10 μm) of polystyrene particles with the same density and compressibility was demonstrated. The applied sample flow rate was varied from 0.03 μL/min to 0.3 μL/min. The ratio of the sample flow rate to sheath flow was kept constant as 1:3. From the analysis of the captured images, it was selected that the optimum flow rates as 0.22 μL/min and 0.66 μL/min for the sample flow rate and sheath flow rate; and the optimum input power as 320 mW, respectively.

In Reference [8], the separation of two 10 μm diameter, different-density particle streams (polystyrene: 1050 kg/m^3^ and melamine: 1710 kg/m^3^) without using sheath flow was demonstrated. The applied input power was ranged from 320 mW to 1280 mW and the flow rate ranged from 0.2 μL/min to 2 μL/min. The separation efficiencies obtained were in the range of 98.8%–89.4%for flow rates ranging from 0.2 μL/min to 2.0 μL/min at 1280 mW input power.

In Reference [9], the separation of two different-size particle streams (3 μm and 10 μm) as well as the separation of two 10 μm diameter with different-density particle streams (1050 kg/m^3^ and 1710 kg/m^3^) was demonstrated. The applied input power was ranged from 300 mW to 1200 mW and the flow rate ranged from 0.1 μL/min to 0.5 μL/min. The captured fluorescent images were used to verify the experimental results.

In Reference [10], input power ranging from 370 mW to 463 mW was applied to separate the blood components. The flow rates of the sample and sheath flow were 0.25 μL/min and 5 μL/min, respectively. As the applied power increased, the RBC clearance ratio also increased. The RBC clearance ratio was over 99% in region of 416–463 mW. On the other hand, the platelets clearance ratio was 74.1% at 439 mW input power.

In Reference [11], the separation two different particle mixtures: fluorescent microspheres of different sizes and human peripheral blood mononuclear cells (PBMCs) mixed with *E. coli* bacteria were demonstrated. The first sample was a mixture of two types of fluorescent microspheres, 1.2 μm in diameter with green emission and 5.86 μm in diameter with red emission. The applied input powers were 85 mW, 240 mW, and 339 mW. The Flow rates of the particle flow and a single side sheath flow were 0.2 μL/min and 0.8 μL/min, respectively. The input power 85 mW was weak to produce sufficient acoustic radiation force. On the other hand, the input power 339 mW was strong to produce sufficient acoustic radiation force. Therefore, the input power 240 mW was used to separate this mixture. The second sample consisted of two different biological cells, purified human PBMCs and *E. coli* bacteria. PBMCs had an averaged diameter around 7.23 μm; while *E. coli* bacteria have an averaged volume similar to a sphere with a diameter of 1.1μm. The applied input power was 468 mW. The flow rates of the cell mixture and sheath flow were 0.5 μL/min and 4 μL/min, respectively. The separation efficiency of *E. coli* and PBMCs were 95.65% and 91.48%, respectively.

As in References [7,8,9,10,11,12], the random high input power, wide range of input power, wide range of flow rates, more than one stage of SSAW, or had low collecting efficiency was used. Also, the design analysis of SSAW microfluidics or the separation analysis was not included in these published papers.

In this paper, The SSAW force with Rayleigh angle effect and its attenuation in liquid-loaded substrate, viscous drag force, hydrodynamic force, and diffusion force are analyzed. Also, the design analysis of SSAW microfluidics for the separation of particles is presented and discussed in details. The design analysis of SSAW microfluidics is depending on three factors. The first factor is the setup properties of the SSAW microfluidics such as: piezoelectric substrate material, input power, wavelength, length of IDTs, width of the main microchannel, widths of outlet collecting microchannels, working area of SAW, working time of SAW, and flow rate of fluid. The second factor is the properties of the fluid such as: density, compressibility, and viscosity. The third factor is the properties of the particles such as: density, compressibility, volume and radius.

The design analysis of SSAW microfluidics is provided for selecting the piezoelectric substrate material, width of the main microchannel, working area of SAW, wavelength, minimum input power, and widths of outlet collecting microchannels required for the separation process. The minimum input power required to shift the maximum volume of the particles to the side wall of the main SSAW microchannel at the end of the working time of SSAW is calculated by simulation programs. The calculated minimum input power required for the separation process is determined with appropriated displacement contrast of the particles. Also, the simulations are provided for minimum power consumption, less complicated setup, and high collecting efficiency compared to other published papers [7,8,9,10,11,12].

The simulation of the separation of blood components (WBCs, RBCs, and platelets) based on the design analysis of SSAW microfluidics is presented. Also, the analysis of SSAW force with Rayleigh angle effect and its attenuation in liquid-loaded substrate, viscous drag force, hydrodynamic force, and diffusion force are presented and discussed.

## 2. The Analysis of Forces and Other Effects Acting in SSAW Microfluidics for the Separation of Particles

The Surface acoustic waves (SAWs) are generated by two IDTs. These SAWs are overlapped and produced SSAW to generate the acoustic radiation force. Therefore, pressure node and antinode are generated. These lead the particles to migrate toward the pressure node or antinode depending on the sign of the acoustic contrast factor (*ϕ*). The acoustic radiation force (*F_SSAW_*) is represented as following [13]:

(3) $$F_{SSAW}=-\left(\frac{\pi p_{0}^{2}V\beta_{m}}{2\lambda }\right)\phi \left(\beta ,\rho \right)\sin\left(2kx\right)$$

The acoustic contrast factor *ϕ*(*β*,*ρ*) can be expressed as following:

(4) $$\phi \left(\beta ,\rho \right)=\left(\frac{5\rho_{p}-2\rho_{m}}{2\rho_{p}+\rho_{m}}-\frac{\beta_{p}}{\beta_{m}}\right)$$

where *p*_0_, *V*, *λ*, *ρ_p_*, *ρ_m_*, *β_p_*, *β_m_*, *k*, and *x* are the acoustic pressure amplitude, the volume of the particle, the wavelength, the density of particle, the density of medium, the compressibility of particle, the compressibility of medium, the wave number, and the distance from the pressure node to the initial particle position, respectively. As shown in Equation (4), the acoustic contrast factor (*ϕ*) is determined by the density and compressibility of the particles and the medium.

The acoustic pressure amplitude (*p*_0_) can be calculated as following:

(5) $$p_{0}=\sqrt{\frac{PZ}{A}}$$

where: *P*, *Z* and *A* are the AC signal power applied to IDT, the acoustic impedance of the piezoelectric substrate, and working area of SAW, respectively. The acoustic impedance of the piezoelectric substrate (*Z*) can be calculated as following:

(6) $$Z=\rho_{s}v_{0}$$

where: *ρ_s_* is the density of the piezoelectric substrate. The power flow in SAW devices can be detected by high sensitive optical measurement [14].

The acoustic contrast factor *ϕ*(*β*,*ρ*) determines whether the particle will move towards the pressure node or the pressure anti-node. If *ϕ*(*β*,*ρ*) > 0, the particles will be moved to the pressure node. If *ϕ*(*β*,*ρ*) < 0, the particles will be moved to the pressure antinode. The most biological particles in liquid medium are moved toward pressure node [15].

The viscous drag force is the resistive force acting on particles in the opposite direction of acoustic radiation force. The viscous drag force is derived from Stoke’s law which describes the motion of sphere particles in a viscous fluid at a specific velocity. The viscous drag force (*F_v_*) is given by Stoke’s law as following [16]:

(7) $$F_{v}=6\pi \eta rv$$

where: *η*, *v*, and *r* are the fluid viscosity, the relative velocity between particles and medium, and the radius of the particle, respectively. The acoustic radiation force is dominant compared to the viscous drag force. Also, the viscous drag force cannot be neglected.

When the particles have constant velocity in the SSAW field, the acoustic and viscous forces are balanced. According to Equations (3), (4), and (7), the time required for the particle to migrate from position *X*_1_ to position *X*_2_ based on SSAW and viscous drag forces only (*t_SSAW_*) can be expressed as following [17]:

(8) $$t_{SSAW}=\left(\frac{3\lambda^{2}\eta r}{\pi }\right)\frac{{\left[\ln\left(\tan\left(\frac{2\pi x}{\lambda }\right)\right)\right]}_{X_{1}}^{X_{2}}}{p_{0}^{2}V\beta_{m}\phi \left(\beta ,\rho \right)}$$

The displacement of particles (*d_SSAW_*) based on the SSAW and viscous drag forces can be approximately expressed as following [15]:

(9) $$d_{SSAW}=\frac{\lambda }{2\pi }\tan^{-1}\left(e^{c}\right)$$

(10) $$c=t_{w}\left(\frac{p_{0}^{2}V\beta_{m}}{3\lambda^{2}\eta r/\pi }\right)+\ln\left(\tan\left(\frac{2\pi x}{\lambda }\right)\right)$$

where: *t_w_* is the working time of the acoustic radiation force on the particle.

**Figure 2:**
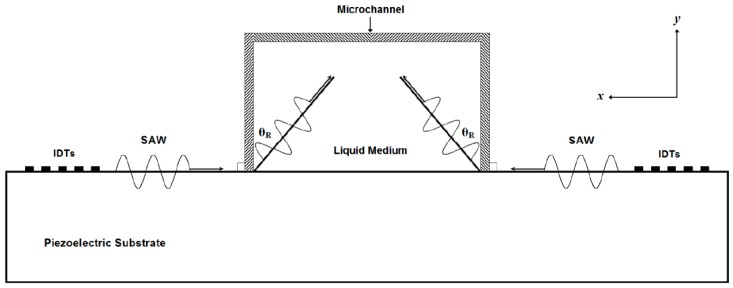
The configuration of Rayleigh angle (*θ_R_*) affect on the propagation of SAW.

The Rayleigh angle (*θ_R_*) is the refraction angle produced due to the changing in SAW propagation velocity in piezoelectric substrate and the liquid medium. The Rayleigh angle (*θ_R_*) can be calculated by Snell’s law as following:

(11) $$\theta_{R}=\sin^{-1}\left(\frac{v_{m}}{v_{0}}\right)$$

where: *v_m_* is SAW velocity in the medium. When SAW meets the edge of the liquid, the energy will couple into the liquid at the Rayleigh angle [18]. The Rayleigh angle is depending on the SAW velocities in the piezoelectric material and the liquid medium. As in [7,8,9,10,11,12], the width of the main microchannel is about *λ*/2 or more. Therefore, the Rayleigh angle can not be neglected. As shown in Figure 2, when SAW propagated from the piezoelectric substrate to the liquid medium, the refracted SAW had horizontal and vertical components. The acoustic radiation force produced by the horizontal component of SSAW is used in the separation process and can be expressed as *F_SSAW_* sin (*θ_R_*).

The attenuation in liquid-loaded substrate affects on the horizontal component of SSAW force. This attenuation is in *x*-direction. The attenuation will also vary in the *z*-direction. The attenuation in *x*-direction is the main important to the separation process; therefore, the attenuation in *z*-direction is not studied in this paper. The horizontal component of SSAW force with the attenuation can be represented as following [19]:

(12) $$F_{\mathrm{SSAW}\;\mathrm{All}}=F_{\mathrm{SSAW}}\sin\;(\theta_{R})\;e^{-2\alpha H}$$

where: *α*, *H* are the attenuation coefficient and path length of SAW in in liquid-loaded substrate. The attenuation coefficient (*α*) can be calculated as following [20]:

(13) $$\alpha =\frac{1}{l_{SAW}}=\frac{\rho_{m}v_{m}}{\rho_{s}v_{0}\lambda }$$

where: *l_SAW_* is the attenuation length of SAW in liquid-loaded substrate. The attenuation coefficient (*α*) is depending on the properties of liquid and piezoelectric material substrate; and the wavelength of SAW. The attenuation is increased exponentially with the attenuation coefficient.

The acoustic radiation force (*F*_SSAW_) with Rayleigh angle (*θ_R_*) effect and its attenuation in liquid-loaded substrate can be expressed from Equations (3) and (11)–(13) as following:

(14) $$F_{\mathrm{SSAW}\;\mathrm{All}}=-\left(\frac{\pi p_{0}^{2}V\beta_{m}}{2\lambda }\right)\phi \left(\beta ,\rho \right)\sin\left(2kx\right)\;\sin\;(\theta_{R})\;e^{-2\alpha H}$$

where: *F*_SSAW All_ is the acoustic radiation force with Rayleigh angle effect and its attenuation in liquid-loaded substrate.

**Figure 3:**
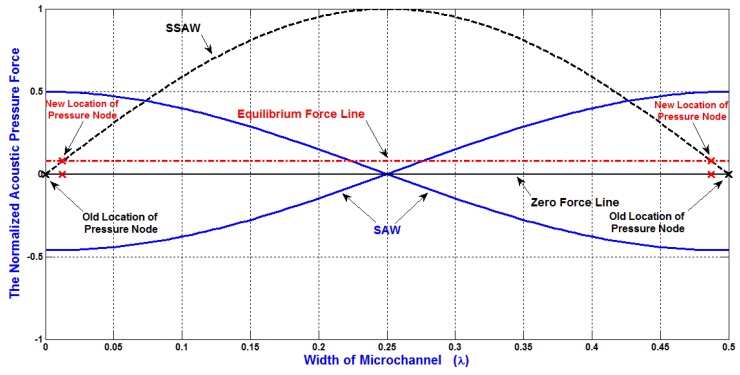
The attenuation effect on the equilibrium force line and the locations of the pressure nodes.

As mentioned previously, Constructive and destructive interferences which result from the two opposing SAWs generate SSAW with the formation of pressure nodes (minimum pressure amplitude) and pressure anti-nodes (maximum pressure amplitude) as shown in Figure 3. These pressure fluctuations result in acoustic radiation forces on the particles in the liquid medium, forcing them migrate toward either the pressure nodes or the pressure anti-nodes, depending on the sign of acoustic contrast factor. The locations of pressure nodes and antinodes are important for the design of SSAW microfluidics to separate the particles.

Without the attenuation effect, the equilibrium force line will be at zero force line. Regarding to the attenuation of acoustic radiation force, the locations of pressure nodes will be affected. As shown in Figure 3, the equilibrium force line is shifted due to the attenuation effect. Therefore, the locations of pressure nodes are changed. As an example, if the width of the microchannel is *λ*/2, without the attenuation effect, the locations of pressure nodes will be exactly on the both sides of the SSAW microchannel. On the other hand, with the attenuation effect, the locations of pressure nodes will be slightly moved toward the center of the SSAW microchannel. Therefore, if the SSAW microfluidics is designed to migrate the particles completely to the side wall of SSAW microchannel (pressure node), the particles will not move completely to the desired locations due to the effect of the attenuation of acoustic radiation force.

The Equation (8) can be modified to express the time required for the particle to migrate from position *X*_1_ to position *X*_2_ based on SSAW force with Rayleigh angle effect and its attenuation in liquid-loaded substrate, and viscous drag force (*t*_SSAW All_) as following:

(15) $$t_{\mathrm{SSAW}\;\mathrm{All}}=\left(\frac{3\lambda^{2}\eta r}{\pi }\right)\frac{{\left[\ln\left(\tan\left(\frac{2\pi x}{\lambda }\right)\right)\right]}_{X_{1}}^{X_{2}}}{p_{0}^{2}V\beta_{m}\phi \left(\beta ,\rho \right)\sin(\theta_{R})e^{-2\alpha H}}$$

The displacement of particle (*d*_SSAW All_) based on SSAW force with Rayleigh angle effect and its attenuation in liquid-loaded substrate, and viscous drag force can be approximately expressed as following:

(16) $$d_{\mathrm{SSAW}\;\mathrm{All}}=\frac{\lambda }{2\pi }\tan^{-1}\left(e^{g}\right)$$

(17) $$g=t_{w}\left(\frac{p_{0}^{2}V\beta_{m}\sin(\theta_{R})e^{-2\alpha H}}{3\lambda^{2}\eta r/\pi }\right)+\ln\left(\tan\left(\frac{2\pi x}{\lambda }\right)\right)$$

The hydrodynamic force is generated due to the flow of the core stream in the inlet microchannel among the flow of symmetric side microchannels. The core stream will be focused in the center of the main microchannel. The assumptions which used to determine the width of the focused stream (*W_f_*) in rectangular microchannelsare explained in details in [5,21]. The width and position of the focused stream can be determined by the relative volumetric flow rates of the three inlets microchannels. The width of the focused stream (*W_f_*) can be calculated using mass conservation law as following [21]:

(18) $$W_{f}=\frac{W_{o}Q_{i}}{\gamma \left(Q_{i}+Q_{S1}+Q_{S2}\right)}$$

where: *Q_i_*, *Q_s_*_1_, *Q_s_*_2_, *W_o_*, *γ* are the volumetric flow rate of the inlet microchannel, the volumetric flow rate of the side microchannels 1 and 2, the width of the main microchannel, and the velocity ratio of the average velocity of the focused stream to the average velocity of the stream in the outlet microchannel which assumed as a unity [22]. The maximum displacement of particle (*d_Hydrodynamic_*) from the center of the main microchannel based on the hydrodynamic force can be calculated according to Equation (18) as following:

(19) $$d_{Hydrodynamic}=\frac{W_{f}}{2}$$

The diffusion force can affect on the separation process. The diffusion is the movement of the solute from high to low concentrations of the solute in the solvent [23]. The particles in the fluid can be moved due to the diffusion force. In general, a small particle diffuses faster than a larger one. According to the simple constant-step random walk model of diffusion, the diffusion displacement can be expressed as following [23]:

(20) $$d_{Diffusion}=\sqrt{Dt_{Diffusion}}$$

where: *d_Diffusion_*, *t_Diffusion_*, *D* are the diffusion displacement, diffusion time, and diffusivity, respectively.

The total displacements of the particles (*d_Total_*) due to SSAW force with Rayleigh angle effect and its attenuation in liquid-loaded substrate, viscous drag force, hydrodynamic force, and diffusion force can be approximately expressed as following:

(21) $$d_{Total}=d_{\mathrm{SSAW}\;\mathrm{All}}+d_{Hydrodynamic}+d_{Diffusion}$$

## 3. The Design Analysis of SSAW Microfluidics and the Simulation of the Separation of Blood Components

The design of SSAW microfluidics is the main factor for successful separation of particles. The design analysis of SSAW microfluidics are not included in the most published papers. The design of SSAW microfluidics for successful separation of particles is depending on three factors. The first factor is the setup properties of SSAW microfluidics such as: piezoelectric substrate material, input power, wavelength, length of IDTs, width of the main microchannel, widths of outlet collecting microchannels, working area of SAW, working time of SAW, and flow rate of fluid. The second factor is the properties of the fluid such as: density, compressibility, and viscosity. The third factor is the properties of the particles such as: density, compressibility, volume, and radius. There are dependant relationships among these three factors. All these factors can affect on the separation process of particles as explained previously.

**Figure 4:**
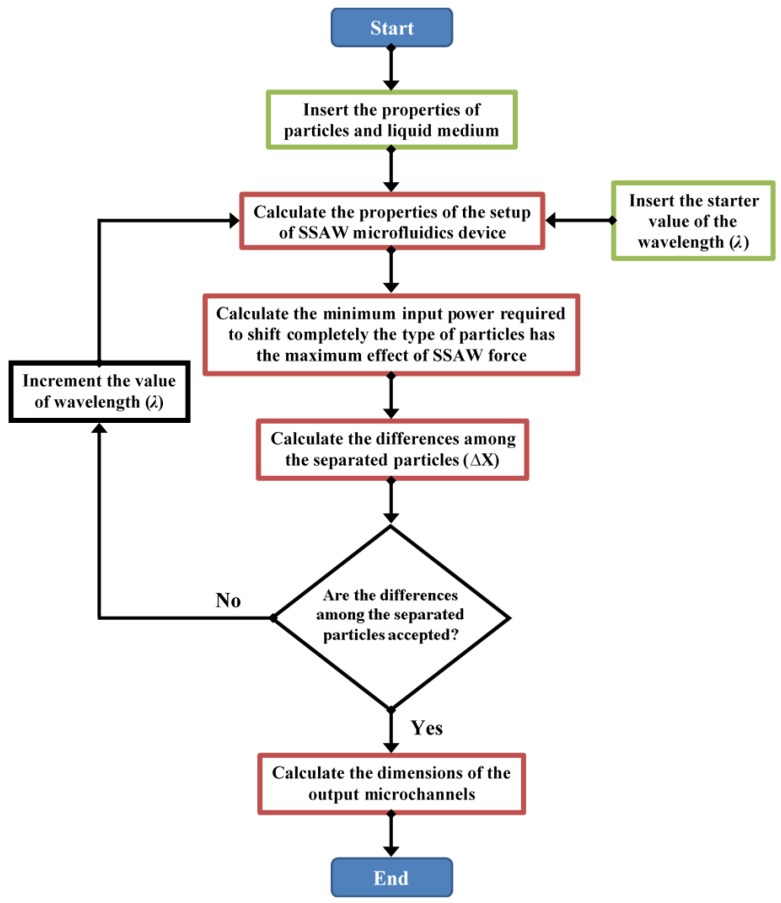
The flow chart of proposed design analysis of SSAW microfluidics for specific separation process.

The flow chart of proposed design analysis of SSAW microfluidics for specific separation process is shown in Figure 4.

The piezoelectric substrate material is important to generate the SAW. The piezoelectric substrate material is the main factor of the value of the horizontal component ratio of SSAW force due to the effect of Rayleigh angle as shown in Equation (11). The horizontal component ratios of SSAW force in distilled water for LiNbO_3_, LiTaO_3_, GaAs, and quartz piezoelectric materials are shown in Table 1. As explained previously, the piezoelectric substrate materials are affecting on the locations of pressure nodes and the attenuation coefficient as shown in Equation (13). The LiNbO_3_ is used as substrate material in the simulations due to its maximum electromechanical coupling coefficient (*K*^2^) compared to the others.

This proposed design analysis is provided to separate the particles types have different properties (size, density, and compressibility).

In the proposed design analysis, the properties of particles and liquid medium are inserted. Also, the starter value of the wavelength (*λ*) is inserted. Then, the properties of the setup of SSAW microfluidics device are calculated based on the mathematical models which described in the previous chapter as following:

1. The width of the main SSAW microchannel is about (2*λ*/5).
2. The wall thickness of the main microchannel is about (*λ*/20).
3. The SAW piezoelectric substrate material is LiNbO_3_.
4. The mixture of components flows in the middle input inlet microchannel as shown in Figure 1.

The lengths of the main SSAW microchannel and IDTs are selected as 15 mm and 9 mm, respectively. The flow rates in the middle input inlet microchannel and the two-symmetric side microchannels are selected as 0.2 μL/min and 0.8 μL/min, respectively. The working time of SSAW force is 0.9 s. The following variables are also calculated:

1. The SAW working area.
2. The Rayleigh angle between the piezoelectric material and the liquid medium.
3. The displacements of particles due to the hydrodynamic force.
4. The displacements of particles due to the diffusion force.
5. The attenuation in the wall thickness of the main microchannel.
6. The attenuation in the liquid medium.

The particle type which has the maximum effects of SSAW force from the center to the side wall of SSAW microchannel is determined. For this particle type, the minimum input power signal required to shift completely to the side wall of SSAW microchannelis calculated.

By calculating minimum input power, the differences among the separated particles (∆Xs) at the end of the working area of SSAW force are calculated.

If the differences among the separated particles (∆Xs) are accepted, the dimensions of output microchannels can be calculated. The separation is accepted under the following conditions:

1. The calculated difference between two types of particles is about (3*R*_1_ + 3*R*_2_). Where: *R*_1_ and *R*_2_ are the radiuses of the first the second particle types, respectively.
2. The width of each output microchannel should be at least 5 times the radius of the particle type.
3. The distance between the calculated position of the particle type and the nearest edge of the output microchannel should be at least 3 times of the radius of the particle type.

The number of output microchannels (*N*_OutputMicrochannels_) can determined as following:

*N*_OutputMicrochannels_ = 1 + (*n* − 1) × 2 (22)

where: *n* is the number of the particles types. The particle type which has the minimum effect of SSAW force will collected from the middle output microchannel. Two-symmetric output microchannels are designed to collect each other particles type.

If the differences among the separated particles (∆Xs) are not accepted, the wavelength (*λ*) will incremented; then, the previous steps will be repeated.

The WBCs, RBCs, and platelets are the main particles in the blood. The values of the density of WBCs, RBCs, and platelets are very closed. Otherwise, the values of the compressibility of WBCs, RBCs, and platelets are the same. Therefore, the volume (radius) is the dominant property affecting on the separation process. The values of radius, density, and compressibility of WBCs, RBCs, and platelets are shown in Table 2 [24,25]. The design analysis of SSAW microfluidics for the separation of blood components is presented.

In our simulation programs, the selection of minimum input power required for the separation process, piezoelectric substrate material, wavelength, width of the main microchannel, widths of outlet collecting microchannels, and working area of SAW are the main target in our design analysis of SSAW microfluidics for the separation of blood components. The constant values of the length of IDTs and working time of SAW are 9 mm and 0.9 s, respectively. The constant values of the ratio of the flow rates of input inlet microchannel to the symmetric side microchannels are used to provide 0.9 s working time of SAW. To simplify the simulation in this study; therefore, the distilled water is used as a fluid carrier of blood components and as sheath fluid. The density, compressibility, and viscosity of distilled water are 1000 kg/m^3^, 448 T·Pa^−1^, and 1 mPa·s, respectively. The simulation programs are built by MATLAB.

In the design analysis, the minimum input power required to shift the maximum volume of the particles to the side wall of the main SSAW microchannel at the end of the working time of SSAW is calculated and used to decrease the power consumption in SSAW microfluidics. The calculated minimum input power has relationships with the properties of particles, fluid, and other properties of SSAW microfluidics.

**Figure 5:**
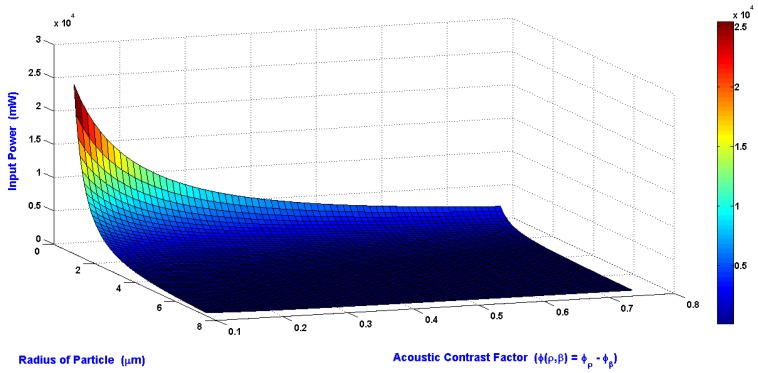
The relations of the input power required to shift the particle to the side wall of the main SSAW microchannel at the end of the working time of SSAW, radius of the particle, and the acoustic contrast factor.

The relations of the minimum input power required to shift the particle to the side wall of the main SSAW microchannel at the end of the working time of SSAW, radius of the particle, and the acoustic contrast factor are shown in Figure 5. The wavelength, the SAW working area, and the piezoelectric material which used in this simulation are 300 μm, 1.08 mm^2^, and LiNbO_3_, respectively. The radius of particle is varying from 1 to 7.5 μm. The acoustic contrast factor is varying from 0.1 to 0.75. The required input power is inversely proportional to radius of particle and acoustic contrast factor.

**Figure 6:**
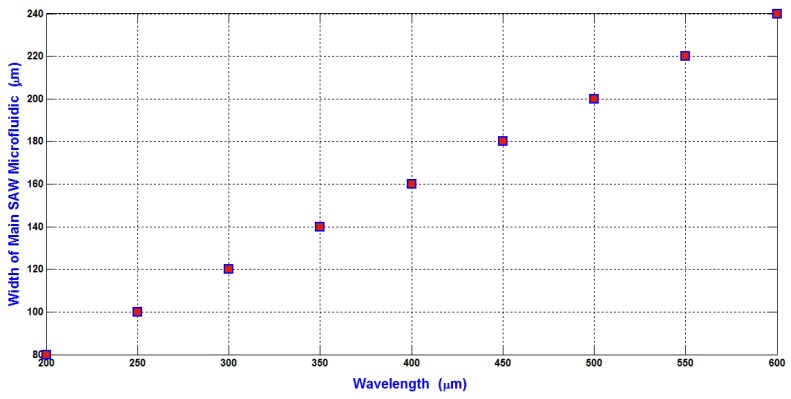
The relation between the width of the main SSAW microchannel and the wavelength.

In our design analysis, the width of the main SSAW microchannel is selected as 2*λ*/5 to avoid the slight moving of the pressure nodes due to the attenuation effect of SAW. Therefore, the relation between the width of the main SSAW microchannel and the wavelength is shown in Figure 6. The width of the main SSAW microchannel is proportional to the wavelength. The working area of SAW is depending on and proportional to the width of the main SSAW microchannel because the length of IDTs is constant as 9 mm. Also, the displacement of particles due to the hydrodynamic force is increased proportional to the width of the main SSAW microchannel as explained previously in Equations (18) and (19) because the ratio of the flow rate of input inlet microchannel to the flow rates of the side microchannels is constant.

**Figure 7:**
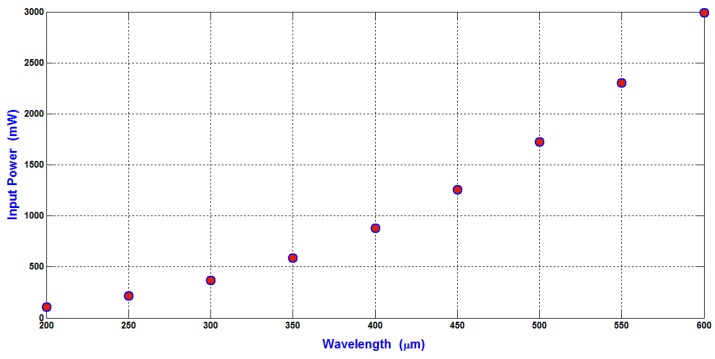
The relation between the minimum input power required to shift the WBCs to the side wall of the main SSAW microchannel at the end of the working time of SAW and the wavelength.

The relation between the minimum input power required to shift the WBCs (the maximum particle compared to RBCs and platelets) to the side wall of the main SSAW microchannel at the end of the working time of SAW and the wavelength is shown in Figure 7.

**Figure 8:**
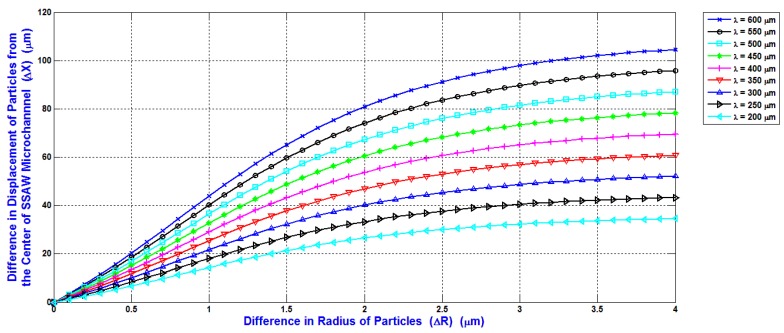
The relations between the difference of particle radius (ΔR) and the difference of the displacement of particle from the center of the main SSAW microchannel (ΔX) at selected values of wavelengths.

The selection of the minimum input power required for separating the blood components WBCs, RBCs, and platelets is the main target in the design analysis. The shifting of the maximum particles (WBCs) to the side wall of the main SSAW microchannel at the end of the working time of SSAW is the reference to calculate the minimum input power. This minimum input power is applied to all blood components. Therefore, the displacement contrast or the difference of the displacement of particle from the center of the main SSAW microchannel (∆X) is essential when selecting the required input power. The relations between the difference of particle radius (∆R) (the reference particle is WBC) and the difference of the displacement of particle from the center of the main SSAW microchannel (∆X) at selected values of wavelengths are shown in Figure 8. For each wavelength in Figure 8, the width of main microchannel and the minimum input power are represented in Figure 6 and Figure 7, respectively. The SAW working time is constant as 0.9 s.

The ∆R between WBCs and RBCs (5 μm–3.5 μm) is 1.5 μm, and the ∆R between WBCs and platelets (5 μm–1.5 μm) is 3.5 μm. At 300 μm wavelength, the displacement contrast (∆X) between WBCs and RBCs is approximately 31 μm, and the displacement contrast (∆X) between WBCs and platelets is approximately 51 μm. From these displacement contrasts, the calculated displacement contrast (∆X) between RBCs and platelets is approximately 20 μm. These displacement contrasts appropriate to optimize the separation process with minimum required input power. Therefore, the 300 μm wavelength is selected for the design analysis. As shown in Figure 7, the minimum input power required for the separation of blood components at 300 μm wavelength is 371 mW.

Most previous SSAW-based devices as in [7,8,9,10,11,12] used random high input power, wide range of input power, wide range of flow rates, more than one stage of SSAW for the separation of particles, or had low collecting efficiency. This separation process can be followed only with high contrast of the properties of particles. The high power consumption, complicated setup, or low collecting efficiency is the disadvantage of these published papers. Also, the separation analysis and the design analysis are not presented in these published papers.

The final SSAW microfluidic design for the separation of blood components (WBCs, RBCs, and platelets) is based on the schematic shown in Figure 1. The most configuration details used in the simulation are collected from [11]. The width and height of the main SSAW microchannel are 120 (2*λ*/5) and 25 μm, respectively. The lengths of the main SSAW microchannel and IDTs are 15 mm and 9 mm, respectively. The SAW wavelength (*λ*) is 300 μm. The SAW piezoelectric substrate material is LiNbO_3_. The flow rates in the input inlet microchannel and the two symmetric side microchannels are 0.2 μL/min and 0.8 μL/min, respectively. The mixture of blood components flows in the input inlet microchannel. The distilled water is used as a fluid. The working time of SSAW force is 0.9 s. The minimum required input power is 371 mW.

**Figure 9:**
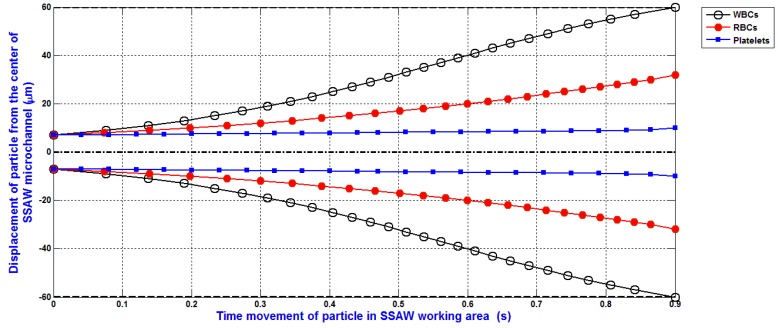
The separation of blood components based on the design analysis of SSAW microfluidics and the analysis of SSAW force with Rayleigh angle effect and its attenuation in liquid-loaded substrate, viscous drag, hydrodynamic, and diffusion forces.

The simulation of the separation of blood components (WBCs, RBCs, and platelets) based on the design analysis of SSAW microfluidics and the analysis of SSAW force with Rayleigh angle effect and its attenuation in liquid-loaded substrate, viscous drag force, hydrodynamic force, and diffusion force is shown in Figure 9. The displacement of WBCs, RBCs, and platelets at the end of the working time of SSAW force due to the SSAW force with Rayleigh angle effect and its attenuation in liquid-loaded substrate, viscous drag force, hydrodynamic force, and diffusion force are 60, 29, and 9 μm, respectively. The separated particles can be simply collected by five outlets microfluidics as shown in Figure 1. Therefore, the widths of the five outlets microfluidics can be determined. The width of middle outlet microchannel for platelets collection is about 30 μm. The width of two mid-lateral outlet microchannels for RBCs collection is about 20 μm. The width of two lateral outlet microchannels for WBCs collection is about 25 μm.

**Figure 10:**
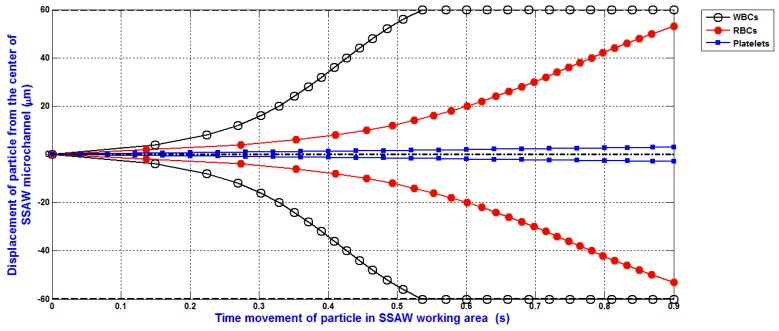
The separation of blood components (WBCs, RBCs, and platelets) based on the design analysis of SSAW microfluidics and the SSAW force only.

The simulation of the separation of blood components (WBCs, RBCs, and platelets) based on the design analysis of SSAW microfluidics and the SSAW force only is shown in Figure 10. This simulation is done under the same simulation conditions of the previous simulation. As shown, the WBCs and RBCs are very closer together (about 7 μm) at the end of the working time of SSAW force. This simulation presents obviously the importance of other forces and effects. The SSAW force only is not enough to provide accurate simulations.

In Reference [10], the separation of platelets from whole blood components (WBCs, RBCs, and Platelets) are demonstrated by input power range from 370 mW to 463 mW. On the other hand, our results represented not only the separation of platelets but also the full separation of whole blood components (WBCs, RBCs, and Platelets) using minimum input power 371 mW and only one separation stage.

These results of the separation of the blood components (WBCs, RBCs, and platelets) demonstrate the design analysis of SSAW microfluidics and the analysis of SSAW force with Rayleigh angle effect and its attenuation in liquid-loaded substrate, viscous drag force, hydrodynamic force, and diffusion force. The analysis study is significant and powerful for the simulation of separation process especially for separation of biological particles.

## 4. Conclusions

This paper demonstrated more accurate theoretical simulation of the separation of particles using SSAW microfluidic devices. The SSAW force with Rayleigh angle effect and its attenuation in liquid-loaded substrate, viscous drag force, hydrodynamic force, and diffusion force were analyzed. The design analysis of SSAW microfluidics for the separation of particles was presented and discussed in detail. The design analysis of SSAW microfluidics was depending on three factors. The first factor was the setup properties of SSAW microfluidics such as: piezoelectric substrate material, input power, wavelength, length of IDTs, width of the main microchannel, widths of outlet collecting microchannels, working area of SAW, working time of SAW, and flow rate of fluid. The second factor was the properties of the fluid such as: density, compressibility, and viscosity. The third factor was the properties of the particles such as: density, compressibility, volume, and radius.

The design analysis of SSAW microfluidics provided the selection of the piezoelectric substrate material, width of the main microchannel, working area of SAW, wavelength, minimum input power, and widths of outlet collecting microchannels required for the separation process. The minimum input power required to shift the maximum volume of the particles to the side wall of the main SSAW microchannel at the end of the working time of SSAW was calculated by the simulation programs and used to decrease the power consumption.

The relations of the minimum input power required to shift the particle to the side wall of the main SSAW microchannel at the end of the working time of SSAW, radius of the particle, and the acoustic contrast factor were presented. The width of the main SSAW microchannel was selected as 2*λ*/5 to avoid the slightly moving of the pressure nodes due to the attenuation effect of SAW. The design analysis of SSAW microfluidics achieved the minimum input power required for the separation process with appropriated displacement contrast of the particles. The relations between the difference of particle radius (∆R) and the difference of the displacement of particle from the center of the main SSAW microchannel (∆X) at selected values of wavelengths were presented.

The final design of SSAW microfluidics for the separation of blood components (WBCs, RBCs, and platelets) was analyzed. The width and height of the main SSAW microchannel were 120 (2*λ*/5) and 25 μm, respectively. The lengths of the main SSAW microchannel and IDTs were 15 mm and 9 mm, respectively. The SAW wavelength (*λ*) was 300 μm. The SAW piezoelectric substrate material was LiNbO_3_. The flow rates in the input inlet microchannel and the two symmetric side microchannels were 0.2 μL/min and 0.8 μL/min, respectively. The mixture of blood components will flow in the input inlet microchannel. The distilled water was used as a fluid. The working time of SSAW force was about 0.9 s. The minimum input power required for the separation of blood components was 371 mW. All simulation programs were built by MATLAB.

The simulation of the separation of blood components (WBCs, RBCs, and platelets) based on the design analysis of SSAW microfluidics was presented. The analysis of SSAW force with Rayleigh angle effect and its attenuation in liquid-loaded substrate, viscous drag force, hydrodynamic force, and diffusion force was discussed and analyzed. The displacement of WBCs, RBCs, and platelets at the end of the working time of SSAW force due to the SSAW force with Rayleigh angle effect and its attenuation in liquid-loaded substrate, viscous drag force, hydrodynamic force, and diffusion force were 60, 29, and 9 μm, respectively. The separated particles were simply collected by five outlets microfluidics. The widths of the five outlets microfluidics were determined. The width of middle outlet microchannel for platelets collection was about 30 μm. The width of two mid-lateral outlet microchannels for RBCs collection was about 20 μm. The width of two lateral outlet microchannels for WBCs collection was about 25 μm.

The simulation of the separation of blood components (WBCs, RBCs, and platelets) based on the design analysis of SSAW microfluidics and the SSAW force only was presented. Under the same simulation conditions, this simulation presented obviously the importance of other forces and effects. The SSAW force only was not enough to provide accurate simulations.

This study provided accurate design and simulations for the separation process. The results of these simulations achieved minimum power consumption, less complicated setup, and high collecting efficiency compared to other published papers. This analysis study was significant and powerful for the simulation of the separation process especially for the separation of biological particles.

## Figures and Tables

**Table 1:** The horizontal component ratios of SSAW force in distilled water for LiNbO_3_, LiTaO_3_, GaAs, and quartz.

Piezoelectric Substrate Material	LiNbO_3_	LiTaO_3_	GaAs	Quartz
The horizontal component ratios of SSAW force in distilled water	0.3745	0.4410	0.5202	0.4565

**Table 2:** The values of radius, density, and compressibility for WBCs, RBCs, and platelets.

Particle Type	Radius (μm)	Density (kg/m^3^)	Compressibility (T·Pa^−1^)
WBC	5	1060	348
RBC	3.5	1096	348
Platelet	1.5	1058	348
